# Nanoparticle delivery enhances the immunomodulatory efficacy of dietary methionine in broiler chickens

**DOI:** 10.1016/j.psj.2026.106436

**Published:** 2026-01-11

**Authors:** Mehran Mehri, Mahmoud Ghazaghi, Morteza Asghari-Moghadam, Mohsen Amraie, Amir Karamzadeh-Dehaghani, Mohammad Rokouei

**Affiliations:** a Department of Animal Sciences, Collage of Agriculture, University of Zabol, Zabol 98661-5538, Iran; b Department of Animal Sciences, Agriculture Faculty, Lorestan University, Lorestan, Iran; c Department of Animal Sciences, Campus of Agriculture and Natural Resources, University of Tehran; Karaj 77781-31587, Iran

**Keywords:** Nano-methionine, Bioavailability, exponential regression, Immunomodulation

## Abstract

Methionine, an essential sulfur-containing amino acid, is fundamental for humoral immunity through its roles in immunoglobulin synthesis and as a precursor to the intracellular antioxidant glutathione. Enhancing the bioavailability of such key nutrients is a significant goal in nutritional immunology. This study investigated whether nanotechnology could enhance the immunomodulatory effects of methionine by comparing the efficacy and relative bioavailability (RBV) of conventional DL-methionine (DL-Met) with a novel nanoparticle-conjugated methionine (nano-Met) in an avian model. The humoral immune response was quantified by measuring primary and secondary antibody titers following challenge with a T-cell-dependent antigen (sheep red blood cells). Exponential regression modeling revealed that the relative bioavailability of nano-Met was 279% (95% CI: 80–479%) compared to DL-Met. Furthermore, dose-response analysis confirmed that a lower concentration of nano-Met was required to achieve a maximal antibody response, indicating superior biological efficiency. These findings demonstrate that nanoparticle-based delivery systems can dramatically increase the bioavailability and immunomodulatory efficacy of essential amino acids. This approach could have significant implications for human health, particularly in clinical contexts requiring enhanced immune function or in populations with compromised nutrient absorption, offering a novel paradigm for targeted nutritional intervention.

## Introduction

The global imperative to ensure food security for a growing population necessitates innovative strategies to maximize nutrient efficiency in both human and animal food systems (Godfray et al., 2010). A significant portion of essential nutrients, such as amino acids, added to diets can be lost due to incomplete absorption, leading to economic inefficiency and increased environmental impact. Nanotechnology is emerging as a transformative platform to address these challenges by fundamentally altering how nutrients are delivered and utilized at a cellular level (Weiss et al., 2006). Through techniques like nano-encapsulation, nanoparticle-based delivery systems can protect nutrients from degradation, improve solubility, and facilitate targeted uptake, thereby enhancing their bioavailability and physiological impact (Shi et al., 2010).

Among the essential nutrients, sulfur-containing amino acids play roles that extend far beyond simple protein synthesis. Specifically, methionine is key for immune function, directly creating immunoglobulins and the main antioxidant, glutathione, which protects immune cells during inflammation (Grimble, 2006). Consequently, optimizing methionine delivery is not just a matter of promoting growth but is fundamental to bolstering health and disease resistance.

The broiler chicken (*Gallus gallus domesticus*) serves as an exceptionally relevant model for this investigation. As a primary source of global dietary protein, enhancing nutrient efficiency in poultry has profound implications for sustainable food production. Moreover, the bird model is established in immunology and nutritional science, reacting sensitively and rapidly to dietary interventions (Garcia et al., 2021; Klasing, 2007). Insights gained from this model can illuminate fundamental biological principles of nutrient absorption and immunomodulation that have translational relevance across species.

While the potential of nanotechnology in nutrition is widely recognized, its specific application to enhance the immunomodulatory efficacy of essential amino acids remains largely unexplored. This study addresses this knowledge gap by investigating the hypothesis that a nanoparticle-conjugated form of methionine (nano-Met) can elicit a superior immune response compared to conventional DL-methionine (DL-Met). We quantify the effects of these two sources on the humoral immune response and determine the relative bioavailability of nano-Met, providing critical insights into the potential of nanotechnology to reshape nutritional strategies for improved health and food production efficiency.

## Materials and methods

### Ethics statement

This study protocol adheres to the guidelines established by the Iranian Council of Animal Care and it has been approved by the Research Animal Ethics Committee (AECUZ-2012-BR) at the University of Zabol.

### Bird management

A total of 825 male day-old Ross 308 broiler chicks were randomly allocated to 11 dietary treatments, each with 5 replicates of 15 birds. The trial lasted 21 days, during which birds were reared in floor pens under controlled temperature and lighting conditions simulating commercial practices Feed intake (FI), body weight gain (BWG), and feed conversion ratio (FCR) from 75 birds per treatment were measured (Ghazaghi et al., 2024).

### Experimental diets

The basal starter diet (Table 1) was based on corn and soybean meal (mesh form) and was marginally deficient in methionine and cysteine (0.35% each), while meeting all other nutrient and energy requirements (Aviagen, 2022). Ten additional diets were formulated by supplementing the basal diet with either DL-methionine (Evonik Degussa GmbH, Germany) or nano-methionine at five graded levels (0.05, 0.10, 0.15, 0.20, and 0.25% of diet), replacing cornstarch. This resulted in 11 diets in total, corresponding to 0.40–0.60% standardized ileal digestible methionine. This approach was intentionally chosen to create a measurable biological response to graded methionine supplementation, thereby increasing the sensitivity of the dose–response model and enabling reliable estimation of relative bioavailability between methionine sources. Feed ingredients containing protein were analyzed for crude protein and amino acid composition (AOAC, 2006). Samples underwent 24-h acid hydrolysis (6 N HCl), with performic acid oxidation for sulfur amino acids and alkaline hydrolysis (BaOH₂) for tryptophan determination.

**Table 1:** Composition of basal diet.

Ingredient	Amount (g/kg)
Corn, Grain	567.5
Soybean Meal-44	309.4
Corn Gluten Meal	61.0
Di-calcium Phosphate	14.9
Oyster Shells	14.0
Corn Starch	10.0
Sunflower Oil	9.20
Sodium Bicarbonate	5.00
L-Lysine HCl	2.80
Mineral Premix^1^	2.50
Vitamin Premix^2^	2.50
L-Thr	1.10
NaCl	0.10
Nutrient specifications	
Metabolizable energy (MJ/kg)^3^	2950
Crude protein (g/kg)^4^	226
Calcium (g/kg)^3^	9.50
Available phosphorus (g/kg)^4^	4.50
SID Met (g/kg)^4^	3.50
SID Cys (g/kg)^4^	3.50
SID Met + Cys (g/kg)^4^	7.00
SID Lys (g/kg)^4^	11.9
SID Arg (g/kg)^4^	12.9
SID Thr (g/kg)^4^	8.30
SID Trp (g/kg)^4^	2.20
SID Val (g/kg)^4^	9.50
DEB (mEq/kg)^5^	250

1 Mineral premix provided per kilogram of diet: Mn (from MnSO4·H2O), 65 mg; Zn (from ZnO), 55 mg; Fe (from FeSO4·7H2O), 50 mg; Cu (from CuSO4·5H2O), 8 mg; I [from Ca (IO_3_)2·H_2_O], 1.8 mg; Se, 0.30 mg; Co (from Co_2_O_3_), 0.20 mg; Mo, 0.16 mg.

2 Vitamin premix provided per kilogram of diet: vitamin A (from vitamin A acetate), 11,500 U; cholecalciferol, 2100 U; vitamin E (from dl-α-tocopheryl acetate), 22 U; vitamin B_12_, 0.60 mg; riboflavin, 4.4 mg; nicotinamide, 40 mg; calcium pantothenate, 35 mg; menadione (from menadione dimethyl-pyrimidinol), 1.50 mg; folic acid, 0.80 mg; thiamine, 3 mg; pyridoxine, 10 mg; biotin, 1 mg; choline chloride, 560 mg; ethoxyquin, 125 mg.

3 Calculated values.

4 Analyzed values.

5 DEB: dietary electrolyte balance represents dietary Na + K – Cl in mEq/kg of diet.

### Humoral immunity assay

To evaluate early post-hatch humoral responsiveness, four birds per replicate were randomly selected, identified using wing bands, and immunized with sheep red blood cells (SRBC). A 5% SRBC suspension was prepared in sterile phosphate-buffered saline (PBS), and each selected bird received 0.1 mL of the suspension via wing vein injection at 7 days of age (primary immunization). The same birds were subsequently re-immunized at 14 days of age (secondary immunization) using the same dose and route. Blood samples (approximately 2–3 mL per bird) were collected from the wing vein at 14 days and 21 days of age, corresponding to 7 days after the primary and 7 days after the secondary immunizations, respectively, to assess primary and secondary antibody responses. Blood was allowed to clot at room temperature and then centrifuged (e.g., 3000 × *g* for 10–15 min) to obtain serum. Serum samples were stored at −20°C until analysis.Antibody titers against SRBC were determined using the hemagglutination inhibition (HI) test, as described by Cheema et al. (2003). Briefly, sera were heat-inactivated (e.g., 56°C for 30 min) and subjected to two-fold serial dilutions in PBS in 96-well microtiter plates. An equal volume of 1% SRBC suspension was added to each well, gently mixed, and the plates were incubated at room temperature until agglutination patterns were clearly visible. The highest serum dilution showing complete agglutination was recorded as the antibody titer. Titers were expressed as the log_2_ of the reciprocal of the highest positive dilution and used for statistical analyses and presentation in the figures.

### Preparation of methionine nanoparticles

Methionine nanoparticles were synthesized by an ultrasonic-assisted method (Aghaei Hakkak et al., 2019). Methionine was dissolved in double-distilled water under constant stirring. Surfactant was added to minimize agglomeration, and the solution was exposed to alternating ultrasonic and microwave irradiation cycles at controlled power. The suspension was centrifuged, and the collected precipitate was dried under vacuum. The nanoparticle preparation method was patented (Patent No: 8463/07/10/1397). As indicated by Ghazaghi et al. (2024), the surface morphology of the nanoparticles was visualized by scanning electron microscopy (SEM). In addition, Fourier transform infrared (FT-IR) spectroscopy (Bruker, Germany) was employed to analyze functional group interactions between DL-methionine and nano-methionine.

### Scanning electron microscopy (SEM)

**Fig. 1:**
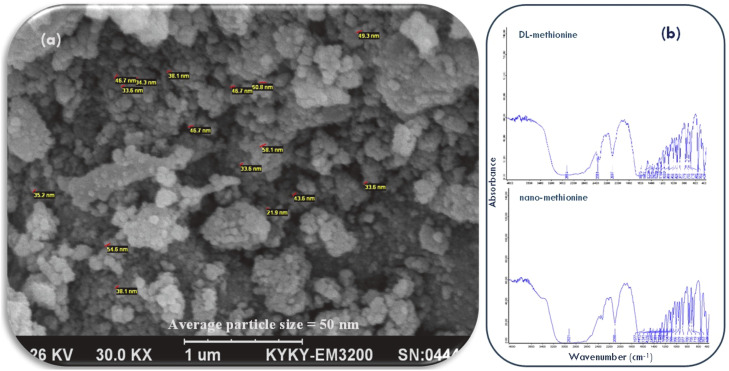
Scanning electron microscope (SEM) of methionine nanoparticles (a); Fourier transform infrared spectroscopy (b); adopted from Ghazaghi et al. (2024).

Scanning electron microscopy was used to evaluate the surface morphology and structural appearance of methionine nanoparticles (Fig. 1a). Prior to SEM analysis, nanoparticle suspensions were first dried to obtain a fine powder. A small quantity of the dried sample was dispersed gently and mounted onto aluminum SEM stubs using double-sided carbon conductive tape to ensure adhesion. Loose particles were removed using mild air flow to reduce clustering artifacts. Because amino acid-based materials are non-conductive and prone to surface charging, the mounted samples were sputter-coated with a thin conductive layer of gold (or Au–Pd) under vacuum. Imaging was performed using SEM operated at a suitable accelerating voltage (approximately 5–15 kV) and working distance optimized for resolution. Micrographs were recorded at different magnifications to assess particle morphology, surface features, and the presence of agglomeration (Goldstein et al., 2017).

### Fourier transform infrared spectroscopy

Fourier transform infrared spectroscopy (FT-IR) was conducted to identify characteristic functional groups and to evaluate possible chemical interactions between DL-methionine and methionine nanoparticles based on changes in absorption band positions and intensities (Fig. 1b). Prior to analysis, both DL-methionine and methionine nanoparticle samples were dried to remove moisture and ground into fine powders to ensure homogeneity. FT-IR spectra were recorded using a Bruker FT-IR spectrophotometer (Bruker, Germany) in the spectral range of 4000–400 cm⁻¹. Measurements were performed at a resolution of approximately 4 cm⁻¹ using multiple scans (e.g., 32 scans) per sample, and the spectra were processed using baseline correction and normalization before comparison. Peak assignments and band shifts were used to interpret functional group interactions and confirm nanoparticle formation (Stuart, 2004).

### Statistical analyses

Data were analyzed as a randomized complete design using the GLM procedure of SAS (2002). Pen means were considered as the experimental unit. Orthogonal polynomial contrasts were applied to evaluate linear and quadratic trends of increasing DL-Met or nano-Met inclusion on antibody response to SRBC.

To estimate methionine requirements, broken-line regression models were fitted Khosravi et al. (2016):

*Two-slope linear ascending -linear descending broken line model:*

Y=L+U×(R−−X)×(X<R)+V×(X−−R)×(X>R)

where Y is the response, L is the intercept, U and V are slopes of the two segments, and R is the breakpoint, interpreted as the nutrient requirement. Model selection was based on the highest R^2^ and the lowest root mean square error (RMSE).

The dose–response relationship was analyzed using a nonlinear regression approach. Model adequacy was first evaluated using ANOVA-based tests for slope linearity, curvature, and equality of intercepts at the basal level (Finney, 1978). Antibody titers against SRBC were used as the primary response variable, and pen means were considered the experimental unit. Bioefficacy of methionine sources was estimated by fitting an exponential response model using the NLIN procedure of SAS/STAT (2002), as commonly applied for nutrient dose–response studies in broilers:

$$Y=a+b\times \left(1-e^{-\left(c\times x_{1}-d\times x_{2}\right)}\right)$$

where *a* = intercept (bird response with basal diet), *b* = scaling factor, *e* = the base of natural logarithms, *c* = steepness coefficient for DL-Met, *d* = steepness coefficient for nano-Met, and x_1_, x_2_ = dietary level of DL-Met, nano-Met, respectively. Relative bioavailability (RBV) of nano-Met compared with DL-Met was calculated as ***d/c***, i.e., the ratio of the exponential slope coefficients, following the regression coefficient ratio approach described by Littell et al. (1997) and previously applied in nutrient bioefficacy evaluation (Mehri et al., 2024).

## Results

As indicated in Table 2 (Ghazaghi et al., 2024), neither supplemental levels of methionine nor methionine sources affected FI. Body weight gain (*P* < 0.001) increased with increasing supplemental methionine, while FCR decreased (*P* < 0.001). Birds fed the basal diet deficient in Met achieved the lowest BWG (28.0 g/b) and the highest FCR (1.69).

**Table 2:** Effects of dietary DL-Met or nano-Met supplementation on feed intake (FI, g/b), body weight gain (BWG, g/b), feed conversion ratio (FCR) in broiler chickens (Ghazaghi et al., 2024).

Response	Basal diet	Experimental diets										SEM	Probability				
		DL-Met					nano-Met										
		0.05	0.1	0.15	0.2	0.25	0.05	0.1	0.15	0.2	0.25		Met	Source	Met × Source	Linear	Quadratic
FI	47.2	46.7	46.5	47.2	48.2	47.1	46.7	46.5	47.6	47.1	46.6	0.64	0.473	0.59	0.915	0.602	0.974
BWG	28.0	29.1	29.6	30.0	30.7	30.3	29.6	29.4	30.5	30.7	30.1	0.58	< 0.001	0.815	0.151	< 0.001	0.042
FCR	1.63	1.61	1.57	1.57	1.57	1.56	1.58	1.58	1.56	1.54	1.55	0.02	< 0.001	0.312	0.828	< 0.001	0.009

**Fig. 2:**
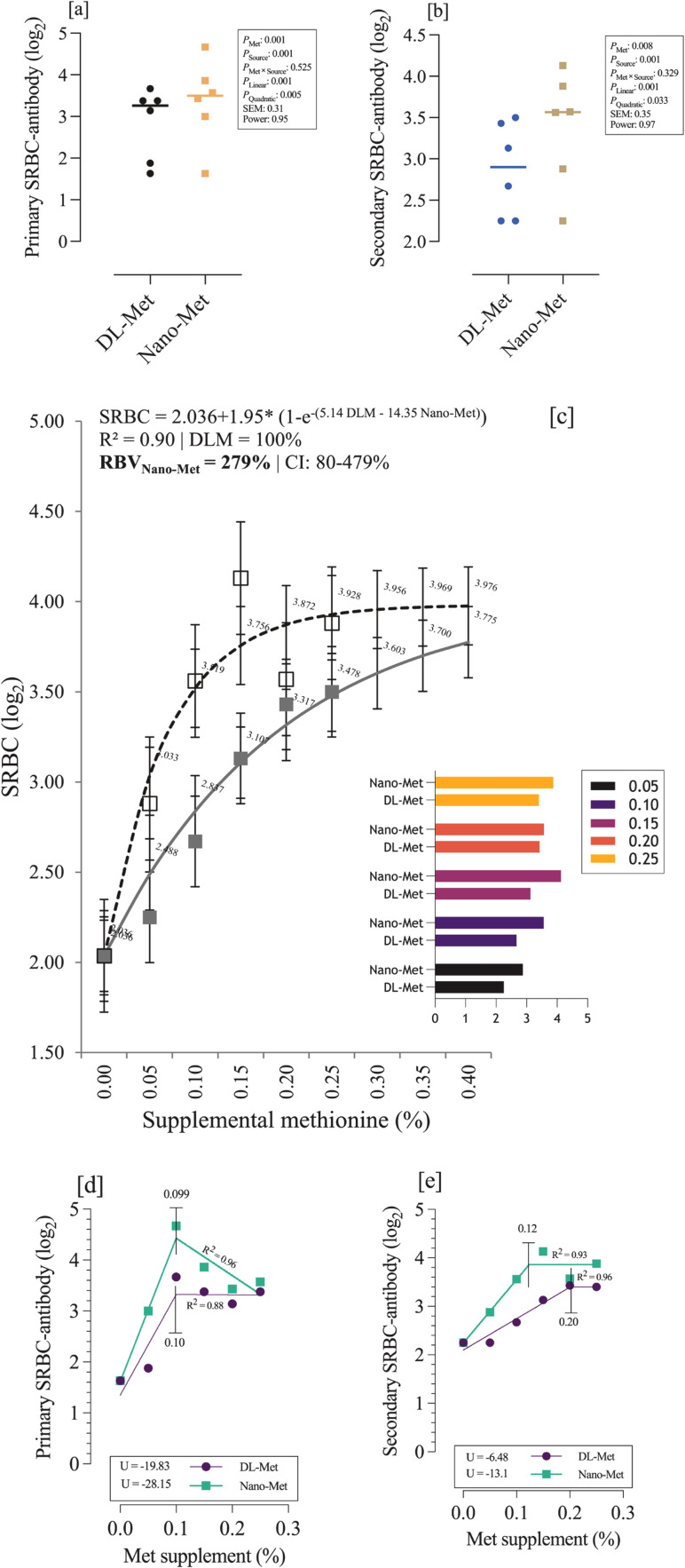
Antibody response to supplemental methionine. Effects of methionine source on primary and secondary SRBC antibody titers (pen means; panels a–b). The statistical significance of supplementation level and source × level interaction is reported in the figure legend (p-values). Relative bioavailability (RBV) of Nano-Met relative to DL-Met estimated using the exponential model (c). Primary and secondary SRBC antibody titers fitted using broken-line regression (d–e).

The antibody response of broilers to SRBC was significantly affected by both the source and level of methionine supplementation (Fig. 2a–b). Primary antibody titers (SRBC I; Fig. 2a) increased with increasing methionine supplementation in both DL-Met and nano-Met treatments (linear *P* = 0.001; quadratic *P* = 0.005). Among the supplemented groups, birds receiving 0.15% nano-Met showed the highest primary response (4.67 log₂), which was markedly higher than the basal diet (1.63 log₂) and the corresponding DL-Met level. Secondary antibody titers (SRBC II; Fig. 2b) showed a similar dose-dependent improvement (linear *P* = 0.001; quadratic *P* = 0.033), with the greatest response observed in birds fed 0.20% nano-Met (4.13 log₂) compared with the basal diet (2.25 log₂).

Statistical evaluation of bioavailability (Table 3) confirmed the relative effectiveness of nano-Met compared to DL-Met. For SRBC I, significant differences were detected in average slopes (*P* < 0.0001), slope difference (*P* = 0.024), blank (*P* = 0.001), intersection (*P* = 0.003), and curvature (*P* = 0.001). For SRBC II, slope difference (*P* < 0.0001) and curvature (*P* = 0.013) were also significant, indicating a superior bioavailability profile of nano-Met over DL-Met in enhancing antibody production.

**Table 3:** Statistical validity of the primary (I) and secondary (II) antibody production against SRBC-antigen challenges for the bioavailability analysis.

Item	Probability (α = 0.05)	
	SRBC I	SRBC II
Average slope	<.0001	0.12
Slope difference	0.024	<.0001
Blank	0.001	0.06
Intersection	0.003	0.18
Curvature	0.001	0.013

SRBC: sheep red blood cell.

Model fitting further supported these findings. Using the exponential function [Fig. 2c], the antibody response to SRBC was described as:

$$SRBC=2.036+1.95\times \left(1-e^{-\left(5.14DL.Met-14.35Nano.Met\right)}\right)$$

with a high fit (*R²* = 0.90). Based on this model, DL-Met was considered the reference (100%), while nano-Met exhibited a relative bioavailability (RBV) of 279% (95% CI: 80–479%).

The broken-line models [Fig. 2d–e] provided estimates of the optimal supplementation levels for each source. For the primary SRBC response, the first slope (U) was −19.83 for DL-Met and −28.15 for nano-Met, with strong fits (*R²* = 0.88 and 0.96, respectively). For the secondary SRBC response, the U values were −6.48 for DL-Met and −13.1 for nano-Met (*R²* = 0.96 and 0.93, respectively). These results suggest that nano-Met requires lower dietary inclusion to achieve maximal immune responses compared with DL-Met, thereby confirming its superior efficiency.

## Discussion

This study provides compelling evidence that nanoparticle-based delivery can dramatically enhance the bioavailability and immunomodulatory function of an essential amino acid. Our central finding, that nano-Met exhibits a relative bioavailability of 279% compared to conventional DL-Met for stimulating a humoral immune response, represents a significant leap forward in nutritional science. The results not only confirm the critical role of methionine in immunity but also demonstrate that its physiological impact can be substantially amplified through nanotechnology, challenging conventional paradigms of nutrient supplementation and efficiency. Although the point estimate suggested a higher RBV for nano-Met, the wide confidence interval (80–479%) overlapped 100%, indicating that the difference in efficacy between nano-Met and DL-Met was not statistically significant; nevertheless, the high upper confidence limit suggests a potentially substantial biological advantage under certain conditions and warrants further confirmation with additional dose levels and a larger dataset.

**Fig. 3:**
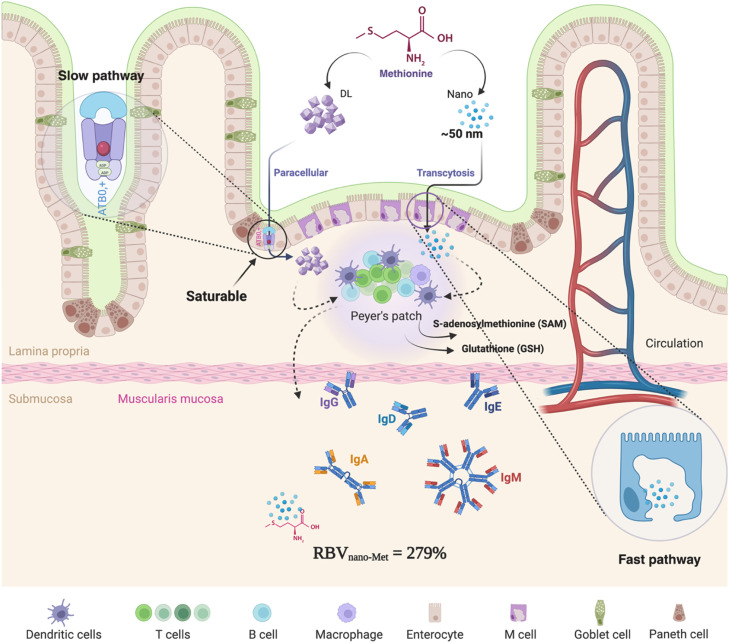
Mode of action of intestinal absorption of DL-methionine and nano-methionine via two mechanisms including transcelluar and transcytosis, respectively (Created in https://BioRender.com).

The superior performance of nano-methionine can be attributed to several potential mechanisms inherent to nanoparticle delivery systems. First, the vast increase in surface-area-to-volume ratio likely facilitates more rapid and complete dissolution in the gastrointestinal lumen, increasing the concentration gradient for absorption (Weiss et al., 2006). Second, nanoparticles may exploit alternative absorption pathways, such as endocytosis or paracellular transport (Fig. 3), bypassing the saturable, carrier-mediated transport systems used by free amino acids (Shi et al., 2010). An important consideration is whether the synthesis process alters the isomeric configuration of the DL-Met precursor. However, it is well-established that avian species efficiently use both d- and l-isomers, with the d-isomer being readily converted to the l-isomer via d-amino acid oxidase and subsequent transamination (Baker, 2009). Thus, any potential changes to the isomeric ratio during synthesis are unlikely to impair the metabolic value of methionine in this model. Elucidating the precise absorption dynamics of nano-methionine is a critical next step for the field.

Based on the available scientific literature, the absorption of conventional DL-Met in broilers primarily occurs through a transcellular transport across enterocytes mediated by specific amino acid transporters. Supplementation with DL-Met has been shown to upregulate the expression of ATB0,+ and B0AT transporters in different segments of the small intestine, supporting this mechanism of active, carrier-dependent absorption (Zhang et al., 2017). Nano-Met may have improved bioavailability because of its small size, enabling transcytosis instead of transporter-mediated uptake. A possible explanation for the enhanced response to nano-Met is that nanoparticles may be absorbed via additional uptake pathways beyond classical amino acid transporters. Previous studies have shown that particulate materials can traverse the intestinal epithelium through microfold (M) cells and may be transported to immune-related sites within the gut-associated lymphoid tissue, including Peyer’s patches, where they can interact with antigen-presenting and other immune cells. Although these uptake mechanisms have been described for nanoparticle systems in general, they were not directly evaluated in the present study. Therefore, this explanation should be considered hypothetical, and further work is required to confirm whether nano-Met follows this pathway and whether it contributes to the higher RBV observed in our experiment. While the M-cell–mediated transcytosis pathway has been well documented for antigen and bacterial particle transport in mammals (Fukuda et al., 2011), direct experimental evidence showing this specific mechanism for nano-Met absorption in broilers remains limited in the current literature.

Our findings extend previous research on the foundational role of methionine in immune function by demonstrating that how this nutrient is delivered is as important as the dose itself. The T-cell-dependent antibody response to SRBC is an exceptionally sensitive endpoint for assessing nutritional impacts on humoral immunity (Ladics, 2007; Tsiagbe et al., 1987). The immunological effects of methionine are multifaceted and center on its conversion to S-adenosylmethionine (SAM), the body's principal methyl donor (Sharma et al., 2025). As the primary source of methyl groups, SAM governs the epigenetic landscape that dictates T-cell fate, controlling the differentiation of naive T-cells into effector lineages and the development of memory T-cells through DNA and histone methylation (Roy et al., 2020).

The principles demonstrated in this study have significant translational potential for human nutrition and therapeutic applications. This nanoparticle delivery platform could be adapted for other essential nutrients, including vitamins, minerals, and other amino acids, to address nutrient deficiencies or support health in vulnerable populations. For instance, it could offer a powerful tool in clinical nutrition for individuals with compromised intestinal function or malabsorption syndromes, or in the formulation of more effective nutraceuticals and functional foods designed to bolster human immune defenses.

While this study provides a strong proof-of-concept, we acknowledge its limitations. Our investigation focused solely on the humoral branch of the immune system, and future work should explore the effects of nano-Met on cell-mediated and mucosal immunity. The long-term physiological impacts, safety, and potential for bioaccumulation of the nanoparticles also require thorough investigation before widespread application. Future research should therefore prioritize elucidating the in-vivo tracking and clearance of these nanoparticles and expanding the toxicological assessments.

It should be acknowledged that the use of a marginally methionine-deficient basal diet (0.35%) was an experimental condition selected to enhance the sensitivity of the biological response to methionine supplementation. This design is appropriate for dose–response modeling and RBV determination, because responses are more pronounced when the basal diet is limiting and the nutrient becomes a primary driver of the measured outcome. However, under nutritionally adequate commercial conditions, the magnitude of response to additional methionine may be smaller as performance and immune traits approach a plateau. Therefore, while the present results support differences in bioefficacy between methionine sources under methionine-limiting conditions, future studies should validate these findings using nutritionally adequate diets to determine whether nano-Met provides an absolute efficacy advantage beyond requirement levels and to assess its practical relevance under typical feeding programs.

Our findings demonstrate that conjugating methionine to a nanoparticle carrier is a highly effective strategy to enhance its bioavailability and immunomodulatory properties. This approach not only offers a powerful tool to improve the efficiency and sustainability of animal food production but also opens new avenues for advanced nutritional interventions in human health. Nanoparticle-mediated nutrient delivery is poised to become a cornerstone of next-generation nutritional science, helping to shape a healthier and more food-secure future.

## CRediT authorship contribution statement

**Mehran Mehri:** Writing – review & editing, Writing – original draft, Formal analysis, Conceptualization. **Mahmoud Ghazaghi:** Writing – review & editing, Resources, Investigation. **Morteza Asghari-Moghadam:** Writing – review & editing, Validation, Data curation. **Mohsen Amraie:** Data curation, Validation, Writing – review & editing. **Amir Karamzadeh-Dehaghani:** Writing – review & editing, Visualization, Software, Funding acquisition. **Mohammad Rokouei:** Writing – review & editing, Resources, Project administration, Methodology, Data curation.

## Disclosures

The authors declare that they have no known competing financial interests or personal relationships that could have appeared to influence the work reported in this paper.
